# Biological Effects of D-Penicillamine on Copper Sulfate-Induced Possible Damage in the Caudate Nucleus: Role of Apoptotic Genes Bax, Bcl2, and Bax-Bcl2 Ratio

**DOI:** 10.1007/s12011-025-04847-z

**Published:** 2025-11-15

**Authors:** Walaa G. Farag, Dina M. M. H. El-Kossi, Esraa K. M. Nafadi

**Affiliations:** 1 Human Anatomy and Embryology Department, Faculty of Medicine, Assiut University, Assiut, Egypt; 2 Physiology Department, Faculty of Veterinary Medicine, Minia University, EL-Minia, Egypt

**Keywords:** CuSO₄, D-penicillamine, Caudate nucleus, Neurons

## Abstract

The role that copper (Cu) plays in encouraging lipid peroxidation reveals the aggressive aspect of copper overload in both human and animal bodies. This process disrupts membrane integrity by generating hydroxyl radicals, leading to increased membrane permeability and uncontrolled leakage of cellular contents, which ultimately contributes to neuronal injury. D-penicillamine, a chelating agent, was used here in our study as its known mechanism is to bind free copper ions and facilitate their urinary excretion. Our point of view is to determine the protective role of D-penicillamine in diminishing the challenges that occurred from over-exposure to copper sulfate (CuSO₄) material on brain tissue, especially at the caudate nucleus (the center of movement and learning in the brain). Thirty adult male albino rats were divided into three groups (10 rats each): control, CuSO₄ (0.2 mg/kg bw), and CuSO₄ (0.2 mg/kg bw) + D-penicillamine (100 mg/kg bw) concurrently once daily for 30 days. We found that CuSO₄ exposure led to oxidative stress, as evidenced by a significant elevation of Malondialdehyde (MDA) and reduction of total antioxidant capacity (TAC), increased levels of interleukin-6 (IL-6) and tissue necrosis factor-α (TNF-α), and raised the Bax/Bcl-2 ratio for provoking apoptosis in brain tissue, which was supported by histopathological examination as marked degenerative changes in the neurons of the caudate nucleus. Apoptotic genes and histopathological images showed a more pragmatic beneficial effect of D-penicillamine on neurons of the caudate nucleus compared to the CuSO₄-treated group. The study highlights the potential role of D-penicillamine as a therapeutic option to counteract CuSO₄-induced toxicity, especially in ecologically affected areas where both humans and animals may be exposed to elevated copper levels.

## Introduction

Caudate nucleus is a bilateral, curved subcortical structure resembling the letter “C,” and it is fundamentally involved in numerous advanced neurological processes. Structurally, each nucleus consists of a prominent front section known as the head, followed by the body, and ending in a slender, tapering tail [1]. As its role in coordinating movement, it is also involved in learning, memory, reward, motivation, emotion, and love relationships. The ipsilateral frontal lobe of the cortex provides the caudate nucleus with input. The thalamus, globus pallidus, and hippocampal regions receive efferent inputs from the caudate nucleus [2]. Caudate nucleus plays a role in stereotyped and repetitive function and as a part of the cortico-striato-thalamo-cortical loop, which is involved in hyperkinetic and hypokinetic movement and mental disorders [3].

Most of the neurons within the caudate nucleus are medium spiny neurons, which primarily function by projecting inhibitory axons to other regions of the basal ganglia. These neurons predominantly utilize gamma-aminobutyric acid (GABA) as their main neurotransmitter [2]. Working memory and executive functioning are influenced by the front region of the caudate nucleus. While the central portion of the caudate nucleus receives input from all areas of the prefrontal cortex, the head of the caudate nucleus and the medial frontal pole have a strong connection. The inferior temporal lobe and the caudate nucleus’ tail work together to analyze visual information and regulate movement. According to Zhang et al*.* [1] certain neurons in the caudate nucleus exhibit selectivity for direction and spatiotemporal correlations which are specific for visual properties.

The biochemistry of entire living things depends heavily on Cu, an important metal. Numerous proteins and enzymes known as cuproenzymes use it as a cofactor. Basic processes like oxygen transport, energy generation, cell metabolism, cell signaling, and hematopoiesis are all impacted by Cu. Additionally, it works particularly well in the mitochondria, which are the cell’s energy-producing organelles, and it contributes to the catalysis of enzymes involved in processes like iron homeostasis, free radical elimination, and collagen and elastin bond creation [4]. According to *Kahveci *et al*.* [5], Cu serves a variety of purposes in cell biology, including signal transmission, angiogenesis, cell migration, and cellular oxidation and reduction events.

If environmentally over-exposed, Cu can be poisonous to both people and animals by irritating the gastrointestinal tract. Also, any disruption in its excretion causes it to build up in tissues and organs [6]. Copperiedus is the term for the excess of Cu in the body, which can be brought on by consuming acidic foods prepared with uncoated Cu utensils or by consuming too much Cu from food, drink, and other environmental sources. Additionally, Cu is widely utilized in electronic devices, steel industries, fertilizers, pesticides, electroplating, and some intrauterine devices [4].

The primary organ in charge of processing and moving extra Cu to be expelled is the liver in its bile, which also stores the majority of Cu. The brain, which can obtain Cu via blood or CSF, is the second vital organ to acquire the most Cu. Genetic mutations resulting in excess Cu cause neurodegenerative disorders such as Parkinson’s, Wilson’s, and Alzheimer’s diseases [6]. Conversely, Menke’s disease, which can be fatal, can be brought on by low Cu levels in the brain [7].

Excess Cu in the body has been associated with the generation of reactive oxygen species (ROS), which contribute to chronic inflammation, oxidative stress in brain tissue, and eventually neuronal cell death. ROS also intensify oxidative damage by altering the structure of proteins, lipids, and nucleic acids, and by triggering nuclear translocation and structural changes [4]. As noted by Erfanizadeh et al. [4] and Elseweidy et al. [7], oxidative stress is considered a key factor in the neurological damage resulting from Cu accumulation.

D-penicillamine is a penicillin derivative without antibiotic activity, despite the fact that its name sounds similar to the well-known antibiotic. It is a well-known chelating agent, a chemical substance that forms a soluble complex that is eliminated from the body by the kidneys in the urine. This complex traps or removes heavy metals like Cu, lead, iron, and mercury by binding free Cu ions. Furthermore, it demonstrates favorable immunomodulatory and antifibrotic properties [8]. Wilson’s disease, heavy metal poisoning, cystinuria, and rheumatoid arthritis are among the conditions that are treated with it in humans because of its metal chelating qualities [9].

The protective effects of D-penicillamine against CuSO₄-induced neuronal damage remain insufficiently understood. Therefore, this study aimed to explore in depth how D-penicillamine may mitigate the harmful impact of excessive CuSO₄ on the caudate nucleus in adult rat brains. The prior research on D-penicillamine has centered on its role as a metal chelator, emphasizing its systemic outcomes. Collectively, the current investigation fills a clear gap in the literature by providing region-specific, mechanism-anchored evidence for penicillamine’s actions in the caudate nucleus as a defined striatal region, thus moving beyond generalized brain or whole-animal endpoints. We assessed inflammatory markers, evaluated oxidative and antioxidant balance, examined the Bax/Bcl-2 apoptotic signaling ratio, and analyzed electro-histopathological changes in the caudate nucleus region.

## Material and Methods

### Chemicals and Drugs

CuSO₄ and D-penicillamine were acquired from Sigma-Aldrich Chemical Co., St. Louis, Mo (USA), and dissolved in distilled water.

### Animals

Thirty male adult albino rats aged 2 months and weighing 170–180 g were used in the experiment. The animals were obtained from the Assiut University Faculty of Medicine’s animal house. Rats were acclimatized for 2 weeks under conventional laboratory conditions prior to any treatments with a standard light/dark cycle and temperature of 25 °C in order to adapt them to the controlled laboratory environment. The rats were maintained on a standard laboratory diet (rat pellets) *ad* libitum purchased from Ibex International Co., Ltd., Egypt (22% protein, 4.10% crude fiber, 2.70% fat, 10% ash, and 61.2% carbohydrate “2800.00 kcal/kg”), with free access to water. This study was conducted after acceptance from the Committee of Animal Research Ethics at the Faculty of Medicine, Assiut University, Assiut (Approval no: 042024300519).

### Experimental Design

Thirty albino rats were divided randomly into 3 equal groups, each containing 10 rats.

- **Group I (Control group):** received distilled water through an intragastric tube once daily for 30 days.
- **Group II (CuSO₄-treated group):** received CuSO₄ by intragastric tube at a dose of 0.2 mg/kg bw once daily for 30 days [6].
- **Group III (CuSO₄ and D-penicillamine-treated group):** received both CuSO₄ (0.2 mg/kg bw once daily for 30 days) and D-penicillamine (100 mg/kg bw) by intragastric tube once daily for 30 days [6].

The selected dose of 0.2 mg/kg bw of the CuSO₄ was chosen based on previous literature and scientific evidence of Fawzy et al. (2022) who have demonstrated its relevance and suitability for investigating the intended biological effects without causing acute toxicity. The investigators also proved that a dose of 100 mg/kg bw of D-penicillamine was potent for the experimental studies [6].

At the end of the experiment, all rats were anesthetized by ether inhalation. In each group, five rats were perfused through the heart with 0.9% saline until blood was fully flushed out, then fixed with 10% neutral-buffered formalin. However, for those intended for electron microscopy, glutaraldehyde was used after saline instead of formalin. The brains of each rat were promptly extracted, and the caudate nucleus was carefully dissected from the brain and used exclusively for all biochemical and molecular analyses. Additionally, following anesthesia, the remaining 5 rats from each group were sacrificed by decapitation, and their brains were immediately collected and prepared for biochemical measures and gene analysis.

### Body and Brain Weights

Body weight was measured for each animal at the end of the experiment before scarification. After exclusion of the brain from each animal, it was weighed to be registered in our experiment.

### Dissection and Homogenization of Tissue Sample

Following the collection of brains from 5 rats from each group, they were divided into two parts: one part for biochemical estimations and pro-inflammatory markers; the second part for mRNA gene expression analysis. The extracted brains were weighed and homogenized in phosphate buffer solution (pH 7.4). The resulting homogenate was centrifuged at 10,000 *g* for 15 min, and the supernatant was collected in aliquots for biochemical analysis and ELISA. For qRT-PCR analysis, brain samples were preserved in RNA later (Qiagen) and stored at −80 °C until further use.

### Total Protein Assessment

Total protein reagent is intended for the in-vitro quantitative and diagnostic determination of total protein in caudate nucleus homogenate.

Animals were sacrificed by decapitation and cortex and basal ganglia samples were collected. Brain samples were homogenized in ten times the volumes of the buffer (50 mM Tris buffer, PH 7.4), and centrifuged for 60 min at 100,000 *g* at 2 °C. The protein concentration of supernatant was quantitated by Colorimetric method (Biuret reagent) (Cat. No.:MG310 001) as guided by the manufacturer protocol. When protein combine with Cu^2+^ in alkaline* pH*, Cu. protein complex was formed. The color intensity is directly proportional to the protein concentration. It is determined by measuring the increase in the absorbance at 546 nm.

### Oxidative Stress Parameters

Oxidative stress markers, specifically MDA and TAC, were measured in brain tissue samples. For that, specific colorimetric test kits for MDA from Bio-diagnostic company, Egypt (catalog numbers: MD 25 29) and catalog numbers: MAES0147 for TAC, which were from Assay Genie Company, 25 Windsor Place, Dublin 2D02VY42, Ireland. The assays were conducted according to the manufacturer’s protocols.

MDA levels were determined by incubating the samples with thiobarbituric acid (TBA) under acidic conditions at 95 °C for 30 min, leading to the formation of a TBA-reactive compound. The absorbance of the resulting colored complex was then measured at 534 nm.

TAC was evaluated by reacting the sample’s antioxidants with a defined amount of hydrogen peroxide (H₂O₂). The antioxidants reduced part of the H₂O₂, and the remaining quantity was determined colorimetrically at 505 nm via an enzymatic reaction that transformed 3,5-dichloro-2-hydroxybenzenesulfonate into a colored product.

### Pro-inflammatory Cytokines (TNF-α and IL-6)

TNF-α levels were quantified using the R&D Systems Quantikine rat TNF-α immunoassay kit (catalog no. E-EL-R0019, Elabscience Biotechnology Inc., USA), following the manufacturer’s instructions.

IL-6 levels were measured according to the manufacturer’s protocol using a kit obtained from PeproTech (USA) (catalog no. E-EL-R0015, Elabscience Biotechnology Inc., USA.

### Real-Time Polymerase Chain Reaction (RT-PCR)

Reverse transcription and quantitative PCR (qPCR) analysis of Bax and Bcl-2 mRNA expression in brain tissue was performed using a one-step assay with the GoTaq® 1-Step RT-qPCR System kit (Promega Corporation, 2800 Woods Hollow Road, Madison, WI 53711, USA; Catalog No. TR118), following the manufacturer’s instructions. The relative quantification of mRNA expression was performed using the Biosystem Software integrated into the Applied Biosystems Real-Time PCR Instruments (Thermo Fisher Scientific, Waltham, MA, USA). Primer sequences for the studied genes are presented in Table 1. The thermal cycling program consisted of the following steps:

1. **Reverse transcription:** 1 cycle at 37 °C for 15 min
2. **RT inactivation/Hot-start activation:** 1 cycle at 95 °C for 10 min
3. **qPCR amplification:** 40 cycles of denaturation, annealing, and extension using gene-specific primers (as shown in Table 1)
4. **Final extension:** 1 cycle at 72 °C for 10 min

**Table 1:** Primer sets of the studied genes in the current study sense antisense accession denaturation, annealing, and extension

	Sense	Antisense	Accession number
Β-actin	5′-TAC AGC TTC ACC ACC ACA GC-3′	5′-GGA ACC GCT CAT TGC CGA TA-3′	NM_031144
Bax	5′-AGG ATC GAG CAG AGA GGA TGG-3′	5′-GAC ACT CGC TCA GCT TCT TGG-3′	NM_017059.2
Bcl-2	5′-TGT GGA TGA CTG AGT ACC TGA ACC-3′	5′-CAG CCA GGA GAA ATC AAA CAG AGG-3′	NM_016993.1

### Light Microscopic Examination

The brain specimens were fixed in 10% neutral buffered formalin, pH 7.4, then were processed for the acquirement of coronal serial paraffin sections, 5 μm in thickness. These sections were subjected to hematoxylin and eosin (H&E) according to Suvarna et al*.* [10] to examine the neuronal architecture of the caudate nucleus.

### Electron Microscopic Examination

The caudate nucleus was isolated from brain tissue by making a coronal section just anterior to the optic chiasma, at the level of the corpus callosum. Small specimens (1 × 1 mm) were taken and fixed in phosphate buffered glutaraldehyde for 24 h and post-fixed in 1% osmium tetroxide for 1 h. Semithin sections (1 μm) were prepared and stained with toluidine blue. Ultrathin sections of 50–60 nm were cut by an ultra-microtome from selected areas and then contrasted with uranyl acetate and lead citrate and were photographed with a transmission electron microscope (Joel-JEM-100 CXII; Joel, Tokyo, Japan) in Assiut University, Electron Microscopic Unit [10].

### Statistical Analysis

SPSS, version 22 (SPSS Inc., Chicago, Illinois, USA) was used for statistical analyses. Data were analyzed using analysis of variance (ANOVA), followed by Tukey’s post hoc test, with a significance threshold set at *p* ≤ 0.05. Results were presented graphically as mean ± standard error of the mean (SEM).

## Results

### Body and Brain Weights

The results of body and brain weights were demonstrated in Table 2. CuSO₄ administration significantly reduced body and brain weights compared to the control group (*p* < 0.05). Co-administration of D-penicillamine with CuSO₄ significantly reduced the deleterious effects of CuSO₄ (*p* < 0.05) and restored brain and body weights to control levels.

**Table 2:** The mean body (g) and brain weights (mg) of the adult rats in the different studied groups

	Control	CuSO_4_	D-pen and CuSO_4_
Body weight (g)	239.50 ± 2.67	181.80 ± 1.66 ^a^	236.80 ± 2.95 ^b^
Brain weights (mg)	2100.30 ± 3.61	1740.00 ± 3.58 ^a^	2095.00 ± 3.48 ^b^

*SE* standard error, *D-pen* D-penicillamine

^a^*p* < 0.001 versus Control group

^b^*p* < 0.001 versus CuSO₄ group

### Effect of CuSO₄ and D-Penicillamine on Total Protein and Oxidant-Antioxidant Status

**Fig. 1:**
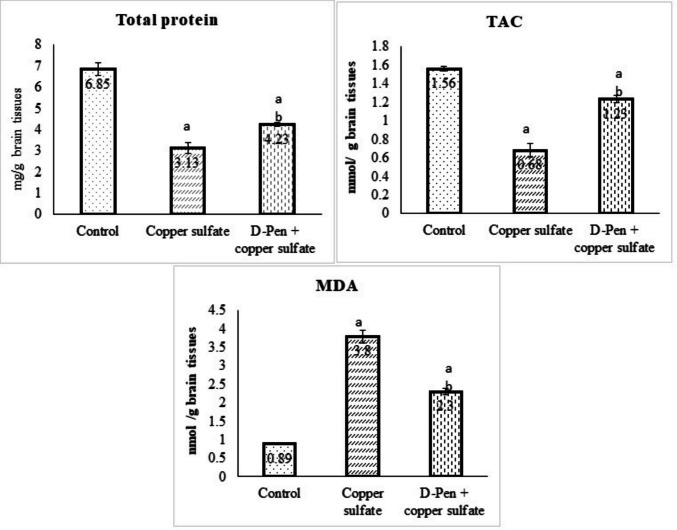
Effect of CuSO₄ and D-penicillamine on total protein and oxidant-antioxidant status (Mean ± SE). SE: standard error, D-pen: D-penicillamine, MDA: Malondialdehyde, TAC: Total antioxidant capacity. ^a^*p* < 0.001 versus Control group. ^b^*p* < 0.01 versus CuSO₄ group

As shown in Fig. 1, CuSO₄ significantly (*p* < 0.001) decreased brain tissue’s total protein levels compared with the control group. Conversely, combining D-penicillamine and CuSO₄ greatly mitigated the negative effects of CuSO₄ and substantially increased total protein levels (*p* < 0.01); however, these levels did not return to control values.

The results in Fig. 1 also revealed that CuSO₄ propagated strong oxidative stress in the brain tissues as evidenced by a significant elevation of MDA and a reduction of TAC (*p* < 0.001). Interestingly, when the rats were co-administered D-penicillamine with CuSO₄, they remarkably raised TAC and lowered MDA relative to the CuSO₄ group (*p* < 0.01).

### Effect of CuSO₄ and D-Penicillamine on Inflammatory Markers

**Fig. 2:**
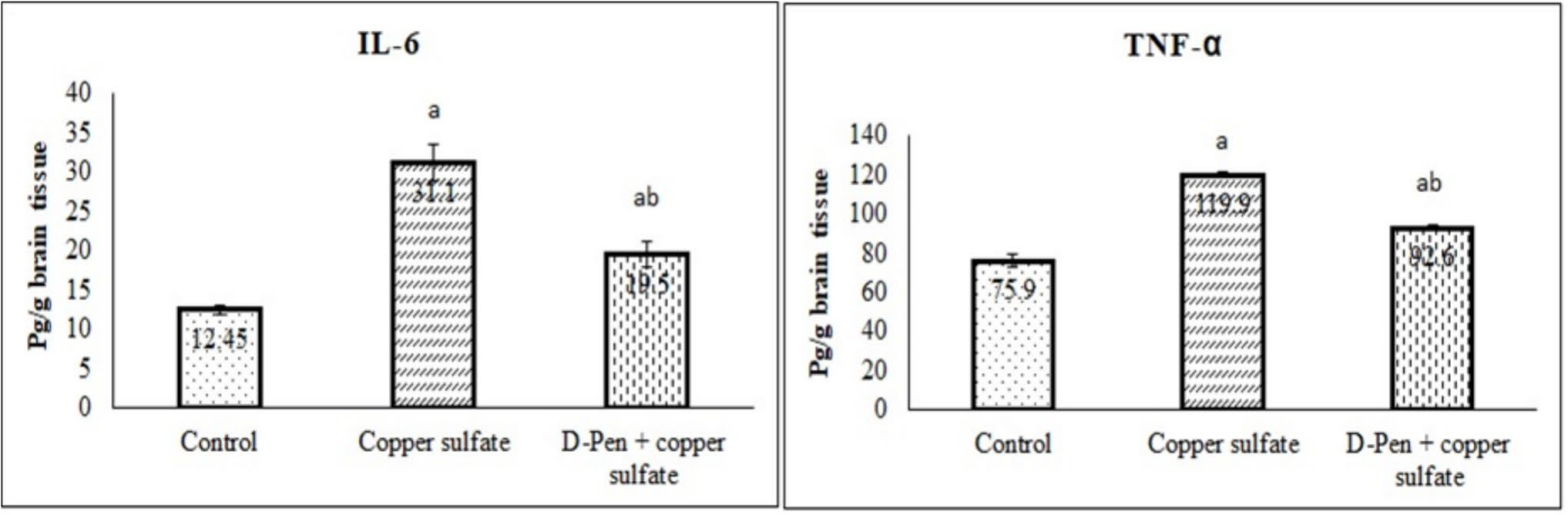
Effect of CuSO₄ and D-penicillamine on inflammatory markers (Mean ± SE). SE: standard error, D-pen: D-penicillamine, IL-6: interleukin-6; TNF-α: tissue necrosis factor-α. ^a^*p* < 0.001 versus Control group. ^b^*p* < 0.01 versus CuSO₄ group

As demonstrated in Fig. 2, CuSO₄ significantly increased the brain levels of IL-6 and TNF-α compared to the control group (*p* < 0.001). On the other hand, D-penicillamine co-treatment markedly reduced (*p* < 0.01) the brain levels of IL-6 and TNF-α that were elevated with CuSO₄.

### Effect of CuSO₄ and D-Penicillamine on Apoptotic Markers

**Fig. 3:**
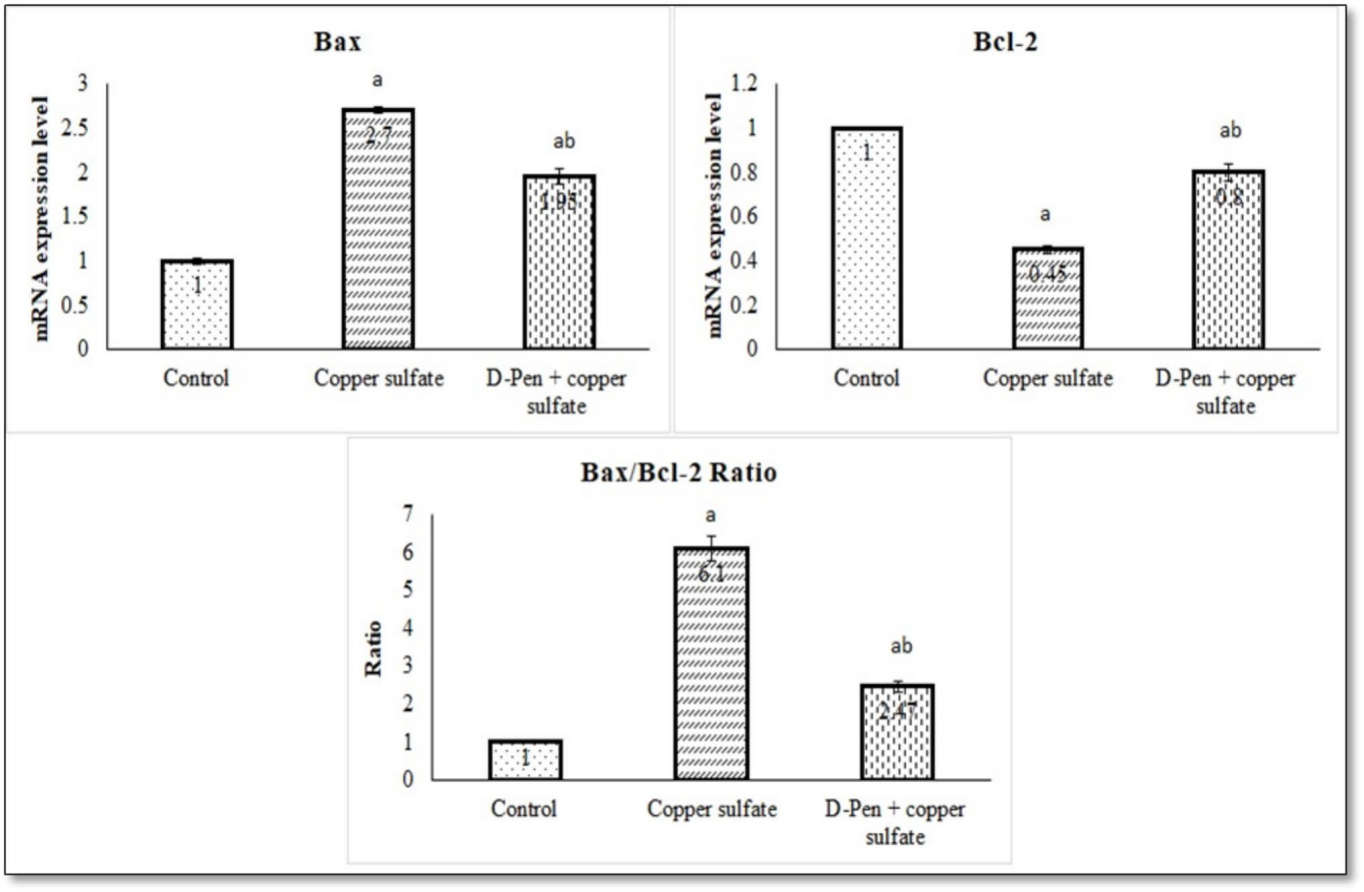
Effect of CuSO₄ and D-penicillamine on apoptotic markers (Mean ± SE). SE: standard error, D-pen: D-penicillamine. ^a^*p* < 0.001 versus Control group. ^b^*p* < 0.01 versus CuSO₄ group

The results in Fig. 3 indicated that CuSO₄ dramatically provoked apoptosis in brain tissues and raised the Bax/Bcl-2 ratio by significantly increasing mRNA brain expression levels of Bax (Pro-apoptotic factor) and decreasing those of Bcl-2 (Anti-apoptotic factor) compared to the control group (*p* < 0.001). The most notable result was that D-penicillamine significantly protected against CuSO₄-induced brain apoptosis and decreased the Bax/Bcl-2 ratio, as the mRNA brain expression levels of Bcl-2 were significantly increased (*p* < 0.01), and those of Bax were significantly decreased.

### Light Microscopic Results Using H&E

**Fig. 4:**
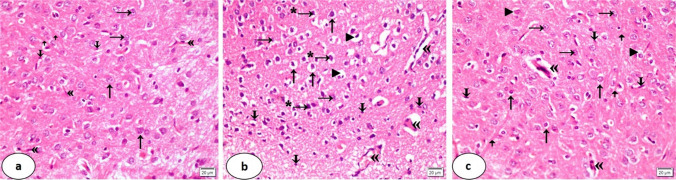
Photomicrographs of a coronal section in the caudate nucleus. Control rats (group I) **(a**) show packed and moderately stained neurons (↑), rounded vesicular nuclei of clear nucleoli (→), some neuroglial cells (ↆ) as well as fiber bundles (ꜛ), normal endothelial lining of the blood vessels («). CuSO₄-treated rats (group II) (**b**) show swollen neurons (↑), rarified nuclei (→). Some nuclei are deeply stained, irregular, and pyknotic (* →). The cytoplasm shows vacuolation (►), many neuroglial cells (ↆ), and absence of fiber bundles. The blood vessels are dilated, congested, and have destructed endothelial lining. They are surrounded by lymphocytic infiltration («). D-penicillamine-treated rats (group III) (**c**) show less swollen neurons (↑), vesicular nuclei of clear prominent nucleoli (→), vacuolated cytoplasm (►) as well as many neuroglial cells (ↆ) and fiber bundles (ꜛ). The blood vessels are dilated and congested («) (H&E stain, × 400; scale bar = 20 µm)

Regarding group I, the neurons of the caudate nucleus were packed and moderately stained. Their nuclei were rounded, vesicular, and had clear nucleoli. Some neuroglial cells as well as fiber bundles were detected within the field. Blood vessels appeared to have normal endothelial lining (Fig. 4a).

In contrast, neurons of group II were swollen. Their nuclei were rarified. Others were deeply stained, irregular, and pyknotic. The cytoplasm had marked vacuolations, and there were many scattered neuroglial cells. The absence of fiber bundles was noticed. The blood vessels were dilated, congested, and showed destructed endothelial lining as well as lymphocytic infiltration (Fig. 4b).

Neurons of group III appeared less swollen. Their nuclei were vesicular and had clear prominent nucleoli. Some neurons showed vacuolated cytoplasm. Many neuroglial cells, as well as fiber bundles, were detected within the field. The blood vessels were dilated and congested (Fig. 4c).

### Light Microscopic Examination Using Toluidine Blue Stain

**Fig. 5:**
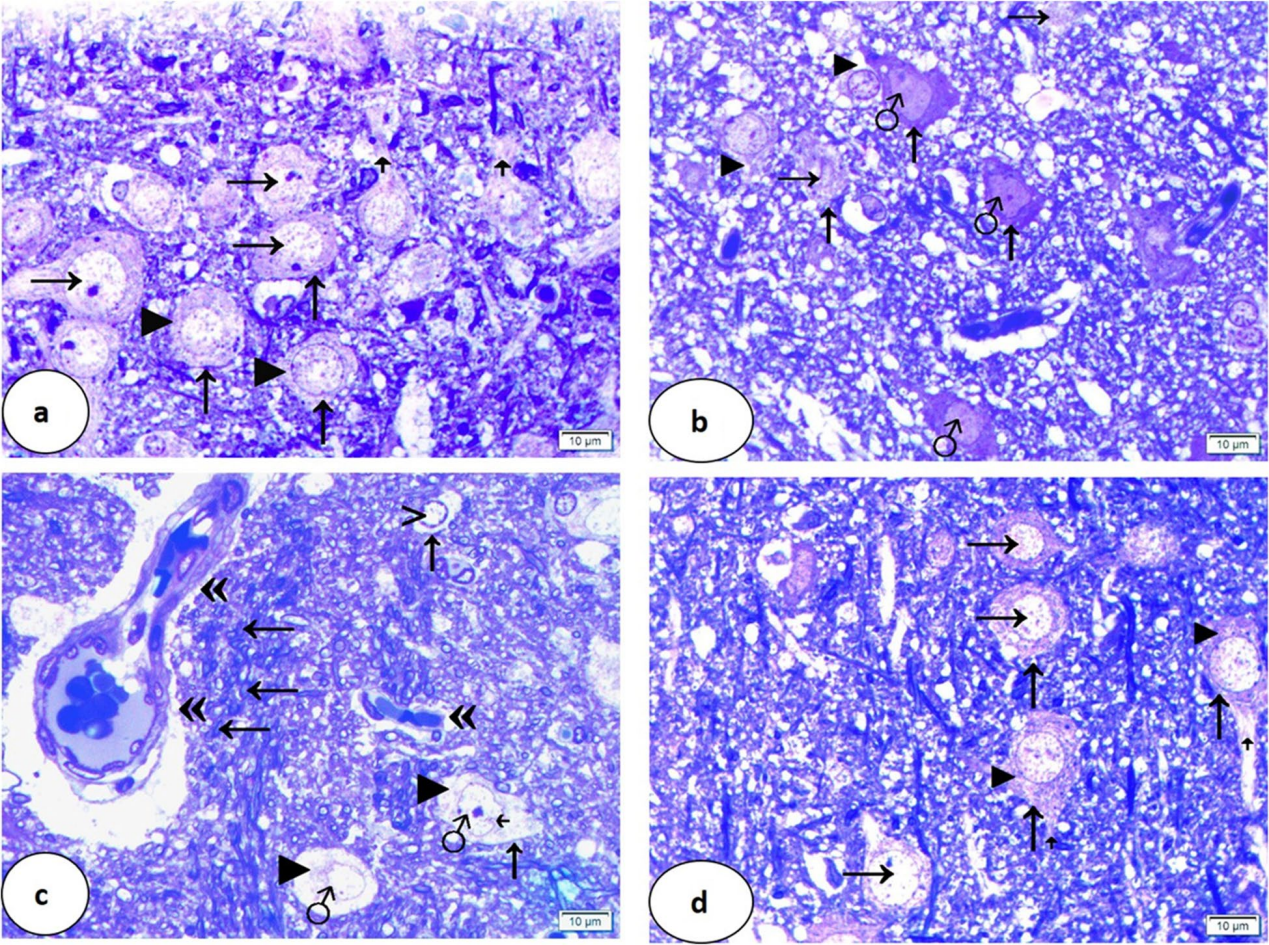
Semithin sections of the caudate nucleus. Group I (**a**) shows closely packed neurons (↑) of large rounded vesicular nuclei and prominent nucleoli (→), granular cytoplasm (►) as well as nerve fibers (ꜛ). Group II (**b**, **c**) shows less packed neurons (↑), rarefied nuclei (→) as well as dark irregular ones (♂), vacuolated cytoplasm (►), and absence of the nerve fibers. In **c**, some neurons show some regular nuclei with peripheral chromatin condensation (>) and obvious indention of some nuclear envelopes (.^˿^). The blood vessels are dilated, congested, and have a destructed endothelial lining («). Marked lymphocytic infiltration around the blood vessels is detected (←). Group III (**d**) shows neurons (↑) of large rounded vesicular nuclei (→), granular cytoplasm (►), and nerve fibers (ꜛ) (toluidine blue × 1000, scale bar = 10 µm)

Regarding group I, neurons of the caudate nucleus were closely packed and showed large rounded vesicular nuclei and prominent nucleoli. The cytoplasm appeared granular (Fig. 5a).

However, neurons of group II were less packed. Nuclei had various changes; some were rarefied, and others were dark and irregular. Some nuclei showed peripheral chromatin condensation. The nuclear envelope of some nuclei showed obvious indention. The cytoplasm appeared markedly vacuolated. The blood vessels were dilated, congested, and had destructed endothelial lining. Marked lymphocytic infiltration around the blood vessels was detected (Fig. 5b, c).

In group III, neurons of caudate nucleus were of large rounded vesicular nuclei and their cytoplasm was granular (Fig. 5 d).

### Electron Microscopic Examination

**Fig. 6:**
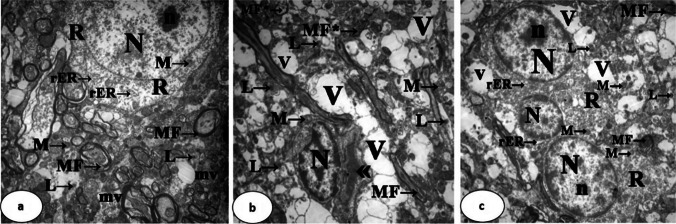
Electron photomicrographs of ultrathin sections in the caudate nucleus. Group I (**a**) shows an oval, euchromatic, regular nucleus (N) with a clear intact double-layered nuclear envelope, a prominent nucleolus (n), cytoplasm containing numerous mitochondria (M →), rough endoplasmic reticulum (rER →), small-sized lysosomes (L →), multivesicular bodies (mv), free ribosomes (R), and regular myelin figures (MF →). Group II (**b**) shows an irregular, shrunken, and heterochromatic nucleus (N), excessively large lysosomes (L →), marked loss of the other cell organelles, vacuolations (V), congested capillaries («), few swollen mitochondria (M →), thin myelin figures (MF →), and fragmented ones (MF* →). Group III (**c**) shows quite regular, rounded, euchromatic nuclei (N) with a clear nuclear envelope, prominent nucleoli (n), marked improvement in the amount of cell organelles such as mitochondria (M →), rough endoplasmic reticulum (rER →), and free ribosomes (R), fewer areas of vacuolation (V), and a smaller number of lysosomes (L →) compared with Group II. Myelin figures are regular and restore their thickness (MF →) (uranyl acetate and lead citrate, × 4800; scale bar = 2 µm)

**Fig. 7:**
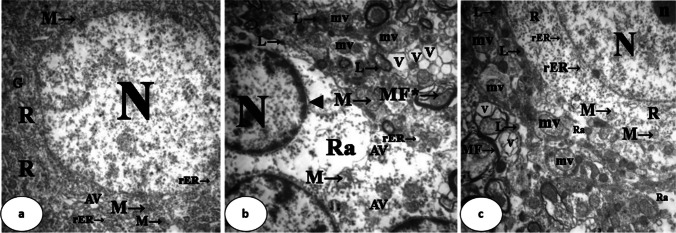
Electron photomicrographs of ultrathin sections in the caudate nucleus. Group I (**a**) shows an oval, euchromatic, regular nucleus (N), numerous cell organelles such as mitochondria (M →), rough endoplasmic reticulum (rER →), free ribosomes (R), alveolate vesicles (AV), and few cisternae of the Golgi apparatus (G). Group II (**b**) shows shrunken nuclei (N), dilated perinuclear cisternae (◄), areas of rarefaction (Ra), multivesicular bodies (mv), many lysosomes (L →), multiple alveolate vesicles (AV), dilated cisternae of rough endoplasmic reticulum (rER →), marked loss of cell organelles, vacuolation (V), swollen mitochondria with destructed cristae (M →), and some fragmented myelin figures (MF* →). Group III (**c**) shows quite a regular, rounded, euchromatic nucleus (N) with a clear nuclear envelope, a prominent nucleolus (n), marked improvement in the amount of cell organelles such as mitochondria (M →), multivesicular bodies (mv), rough endoplasmic reticulum (rER →), free ribosomes (R), and excess lysosomes (L →). Fewer areas of vacuolation (V) and rarefaction (Ra). Myelin figures appear thick (MF →) (uranyl acetate and lead citrate, × 7200; scale bar = 2 µm)

Regarding the ultrastructure of neurons of the caudate nucleus in group I, the nucleus was oval, euchromatic, regular in shape, and of clear intact nuclear envelope. The nucleolus was prominent. The cytoplasm demonstrated numerous mitochondria, rough endoplasmic reticulum, small-sized lysosomes, multivesicular bodies, free ribosomes, alveolate vesicles, and few cisternae of the Golgi apparatus. The nerve fibers were wrapped with a regular myelin sheath forming myelin figures (Figs. 6a and 7a).

In contrast, the nucleus in group II was irregular, shrunken, and heterochromatic. Some were surrounded by dilated perinuclear cisternae. The cytoplasm showed areas of rarefaction, multivesicular bodies, and multiple alveolate vesicles. Rough endoplasmic reticulum showed dilated cisternae. The field had excess large lysosomes as well as a marked loss of the other cell organelles, vacuolations, and congested capillaries. Few mitochondria appeared swollen and had destructed cristae. Some myelin figures appeared thin, while others showed fragmentation (Figs. 6b and 7b).

The ultrastructure of neurons in group III demonstrated quite regular, rounded, euchromatic nuclei of clear nuclear envelope. Their nucleoli were prominent. The cytoplasm showed marked improvement in the amount of cell organelles such as mitochondria, rough endoplasmic reticulum, and free ribosomes. Fewer areas of vacuolation and rarefaction as well as a smaller number of lysosomes were detected within the field as compared with group II. Myelin figures were regular and restored their thickness (Figs. 6c and 7c).

## Discussion

Heavy metals such as Cu are essential to the body’s function in very small amounts. If it accumulates in the body in concentrations sufficient to cause poisoning, a disturbance of the metabolic activities all over the body would take place,as postulated byZangiabadian et al. [11] and Kahveci et al. [5]. Agboolaet al*.* [12] pointed to the capability of Cu to penetrate the blood-brain barrier, where it elicits its neurotoxic effects. D-penicillamine is a beta-dimethyl analog of the amino acid cysteine, chelating with heavy metals to increase their urinary excretion in patients with Wilson’s disease, rheumatoid arthritis, and cystinuria [13].

The current study was designed to examine the protective role of D-penicillamine against Cu-induced brain injury. Group II demonstrated a statistically significant decrease in the body weight as compared to group I. This was in agreement with Quan et al. [14].

The direct systemic toxicity brought on by the Cu might be a cause of the observed body weight differences. Jomova et al. [15] pointed out that Cu is well known to be a hepatotoxicant and nephrotoxicant [16]. This was previously documented by Glicklich et al. [17] who pointed to the concomitant association of the heavy metal toxicity and both chronic hepatic and renal failure. With such organ failure, a subsequent reduction in body weight in the present work could be illustrated.

Additionally, Quan et al*.* [14] reported that Cu exposure compromises the structural integrity of the intestinal epithelium and may alter the composition of the intestinal microbiota in rats. These microbial communities are essential for preserving the intestinal barrier, facilitating nutrient absorption, and supporting immune function [18]. By alteration of the abundance and diversity of the microbial community, the absorption of nutrients could be affected, which explains another possible mechanism for the decrease in body weight in the present work.

Moreover, excessive Cu exposure may encourage disturbances in the mineral equilibrium. This might cause the lipids in the membrane to peroxide, which could alter the membrane’s permeability and cause the fluidity of the body to decrease, which would ultimately result in weight loss [19].

In the present work, the improvement in body weight of group III compared to group II could be a direct result of the protective effect of the penicillamine as postulated by Delle Cave et al*.* [20]. By speeding up the repopulation of the cells, penicillamine enhances the hepatic and renal function [21]. Regaining the lost body weight in the present study might be facilitated by decreasing glycogenolysis and improving renal damage since the process of liver and kidney regeneration improves its functionality as mentioned by Pop and Grama [22].

Regarding the brain weight, group II demonstrated a statistically significant decrease in the brain weight as compared to group I. This was in accordance with Witt et al. [23]. The researchers reported that CNS is a major target tissue of the Cu toxicity. Through the mitochondrial impairment and induction of oxidative stress, functional impairment in the cells takes place. Even through displacing essential metals from their natural binding sites, Cu could bind to the protein regions, leading to the eventual poisoning of the cells. In consequence, DNA damage and the death of the neurons are the net result, as discussed by Jomova et al*.* [15]. With such neuronal tissue loss, the decrease in brain weight of group II in the current work could be explained.

Yang et al. [24] reported that D-penicillamine improved neuronal survival in a mouse model of neurotoxicity. Furthermore, it was validated to suppress the accompanied sequel of neuronal damage. Through such mechanisms, the improvement in brain weight of group III could be clearly understood.

CuSO₄ administration led to a significant reduction in total protein content in brain tissue reflecting Cu’s known toxicological effects. This reduction highlights the disruption of cellular homeostasis, as Cu interferes with protein synthesis and stability. Beyond Cu transport, Cu also exhibits ferroxidase activity, converting Fe^2^⁺ to Fe^3^⁺, thus preventing iron-mediated oxidative stress [30]. In the brain, Cu is synthesized locally in astrocytes and dopaminergic neurons, facilitating iron efflux and maintaining redox balance [31]. The observed reduction in total protein content likely reflects the combined effects of disrupted Cu function and widespread oxidative protein damage induced by excess Cu and ROS.

Treatment with D-penicillamine resulted in a partial but notable restoration of total protein content in brain tissue. D-penicillamine binds free Cu ions, effectively limiting their catalytic role in oxidative reactions and reducing ROS-mediated cellular injury. By lowering bioavailable Cu, the chelator helps preserve the integrity of structural and functional proteins, mitigating the neurodegenerative impact of Cu overload. The partial restoration of protein levels observed in this study highlights the compound’s ability to attenuate oxidative stress and support the maintenance of protein homeostasis in neural tissues. These findings confirm the dual pathological mechanisms of Cu toxicity, metal accumulation, and oxidative protein degradation and suggest that timely chelation therapy can provide significant, though incomplete, neuroprotection [32].

As previously mentioned, Cu exerts its cytotoxic effects primarily by enhancing the production of ROS and disrupting antioxidant defense mechanisms. This is evidenced by increased levels of intracellular MDA, a key marker of lipid peroxidation [25]. This oxidative imbalance may compromise the integrity of the blood–brain barrier. This disruption permits the infiltration of neurotoxic plasma constituents, blood cells, and pathogens into the brain tissue, which causes increased ROS generation, mitochondrial dysfunction, and inflammation. Collectively, these variables cause protein modification, lipid peroxidation, DNA damage, and, eventually, brain cell death [26]. The current results demonstrated that CuSO₄ administration significantly disrupted the oxidant-antioxidant balance in the brain, particularly within the caudate nucleus of rats. This disruption was evidenced by a marked increase in MDA levels along with a significant reduction in TAC, indicating elevated oxidative stress. These findings align with previous research showing that Cu toxicity enhances oxidative damage by promoting lipid peroxidation and impairing endogenous antioxidant defenses [12, 27]. Collectively, our results supported the suggestion that CuSO₄ exerts neurotoxic effects in part by inducing oxidative stress, thereby compromising neuronal integrity in the caudate nucleus.

The administration of D-penicillamine, a Cu-chelating agent, partially restored this redox imbalance. D-penicillamine-treated rats exhibited lower MDA levels and improved TAC, suggesting effective sequestration and excretion of excess Cu. In agreement with the current findings, D-penicillamine supports cellular health by reducing free radical levels through chelation of metal ions—particularly Cu—that promote lipid peroxidation [28]. D-penicillamine forms stable, water-soluble complexes with Cu ions, promoting their renal elimination and attenuating oxidative damage. Furthermore, it reversed the effects of Cu-induced catalase activity suppression and total thiol level decline [29]. These observations underscore the therapeutic value of D-penicillamine in alleviating Cu-induced oxidative burden.

In the present study, we evaluated the impact of CuSO₄ toxicity on inflammatory processes within the caudate nucleus of adult male rats. Our findings demonstrated that Cu accumulation led to a significant increase in pro-inflammatory markers, indicating an active neuroinflammatory response. Consistent with our results, the study of Adeleke et al. [33] found that administering mice CuSO₄ daily for 28 days resulted in an increment of TNF-α and IL-6 in brain tissues. Cu significantly caused the inflammatory activation in brain tissue cells [34] and hepatic tissue [35]. Similarly, findings of prior research showing that Cu buildup in the striatum of mouse models has been associated with increased expression of pro-inflammatory cytokines, with the intensity of the inflammatory response correlating directly with the degree of Cu deposition [36]. These data collectively support the notion that Cu dyshomeostasis can drive neuroinflammatory processes and may contribute to neurodegeneration.

Importantly, we showed the therapeutic efficacy of D-penicillamine in mitigating CuSO₄-induced neuroinflammation in the caudate nucleus. Consistent with our findings, D-penicillamine has been observed to decrease serum levels of cytokines such as IL-1 and rheumatoid factor [37]. As a potent Cu chelator, D-penicillamine binds free Cu ions and enhances urinary excretion, thereby reducing the bioavailable Cu pool that drives oxidative stress and inflammation [38]. This chelation markedly attenuated the levels of inflammatory markers and helped restore redox balance by limiting ROS generation. These findings are consistent with the established use of D-penicillamine in Wilson’s disease and suggest a broader neuroprotective role against Cu-induced CNS toxicity [39]. Previous studies have demonstrated that D-penicillamine therapy in mice reduces hepatic inflammation, normalizes endoplasmic reticulum stress-related gene expression, and corrects epigenetic disruptions such as global DNA hypomethylation [40].

It is commonly established that oxidative stress induces apoptosis. Apoptosis, also known as programmed cell death, is a natural cell death process that is required for appropriate growth and equilibrium in all multicellular organisms [41]. Kabak et al. [42] demonstrated that copper sulfate overabundance promotes apoptosis in several tissues. The mitochondrial apoptosis route is an inherent apoptosis process that contributes significantly to cell death. This pathway’s main components include B-cell lymphoma-2 (Bcl-2) family proteins, mitochondrial pro-apoptosis proteins (Bax), and caspases. The Bcl-2 family of proteins, consisting of both anti- and pro-apoptotic members, serves as a key intracellular regulator of the apoptotic pathway, with the balance between antagonists (Bcl-2, Bcl-xL, Mcl-1, A1) and agonists (Bax, Bak, Bad) determining whether cells undergo apoptosis. These proteins act mainly at the mitochondria, where they modulate mitochondrial membrane potential (MMP). As central integrators of death signals, mitochondria release apoptogenic factors such as cytochrome c, Smac/DIABLO, apoptosis-inducing factor, and endonuclease G from the intermembrane space in response to pro-apoptotic stimuli, thereby initiating programmed cell death [45].

Herein, CuSO₄ provoked apoptosis in brain tissues and raised the Bax/Bcl-2 ratio. Cu accumulation may induce cell death, which is the hallmark of neurodegenerative disease [43]. In this respect, Ren et al. [44] found that protein levels of Bax, Bak, and Bim were markedly increased while those of Bcl-2 and Bcl-xL were markedly decreased. However, by binding to Bak or Bax, the antiapoptotic Bcl-2 and Bcl-xL have been shown to limit apoptosis by blocking the release of cytochrome c and the consequent activation of caspase [45].

Intriguingly, our findings showed that the elevated Bax/Bcl-2 ratio caused by CuSO₄ was remarkably amended by D-penicillamine co-administration. Consistent with our findings, D-penicillamine markedly suppressed the rhododendrol-dependent cell death [46]. D-penicillamine has also been shown to significantly inhibit neuronal ferroptosis by its potential to decrease ACSL4 and COX-2 expression levels [24]. The anti-apoptotic effect of D-penicillamine may be ascribed to its antioxidant properties, which safeguard the brain from oxidative damage, hence preventing mitochondrial dysfunction, cytochrome c release, and Bax-mediated apoptosis.

Light microscopic examination of the caudate nucleus sections in group II (CuSO₄-treated group) demonstrated several degenerative signs in the morphology and architecture of its neurons. These findings were in accordance with Elseweidy et al*.* [7] who recorded similar observations in their study of Cu toxicity in the hippocampus. The researchers reported an increase in inflammatory responses and generation of ROS and considered the oxidative stress a leading cause of apoptosis. Added to that, Kahveci et al. [5] reported that Cu toxicity resulted in a reduction in the total number of neurons in a dose-dependent manner.

Investigators reported that Cu interacts with hydrogen peroxide to generate hydroxyl radicals—highly reactive species capable of damaging vital macromolecules, including membrane proteins and lipids, DNA, and RNA. Also, Cu has been shown to cause several biochemical cascades through oxidative stress that significantly result in neuronal death [12]. Furthermore, a study of *Arowoogun *et al*.* [47] found that the neurotoxic effect of Cu involved GSH depletion, which was implicated in neurodegenerative conditions. The reduction in the length of the nerve fibers was described also as one of the signs of the neuronal damaging effects of Cu [5].

The present study of group II demonstrated rarified pyknotic nuclei. These findings were the basic histological hallmarks of degeneration and come in line with the study of Chen et al*.* [48]. The researchers considered the Cu-induced cell death an important process mediating pathogenesis and the promotion of the degenerative fate of the neurons. Moreover, Chen et al. [49] documented through their studies that disruption of cellular homeostasis could lead to impaired cellular function, with a direct impact on cellular components. This could explain the observed vacuolation of the cytoplasm in the present work.

Theophanous et al. [50] previously reported that neuroglial cells were key regulators in the neuroinflammatory cascade. Neurotoxins, neuronal debris, and injury induce activated microglia that release a range of inflammatory cytokines, whose buildup has harmful effects on neurons and, in turn, triggers further microglial activation, creating a self-perpetuating inflammatory cycle [51]. Persistent microglial activation results in sustained secretion of proinflammatory mediators with subsequent progression of the neuronal damage. This could be detrimental to the nervous tissue, as discussed by Muzio et al. [52]. Moreover, Zhao et al. [53] clarified that excess Cu exposure upregulates the secretion of the inflammatory mediators.This could illustrate the obvious increase in the neuroglial cells in the current study of group II.

The impaired, dilated, and congested blood vessels observed in group II of the current investigation might indicate the presence of associated vascular angiopathy. Zhang et al. [54] ascribed these signs to the inflammatory cytokines generated due to Cu overload in neuronal tissue. In the present study, the possible induced oxidative stress could then be linked to impaired endothelial function and leukocyte adhesion as described by Scioli et al. [55]. The perivascular lymphocytic infiltration of the present work supports the neuroinflammatory theory caused by the Cu toxicity as reported by Liu et al. [56].

Indentation of the nuclei observed in the current results of group II could reinforce the possibility of the coexistence of transient openings in the nuclear membranes of the neurons caused by the toxicity. The nucleocytoplasmic barrier then might allow uncontrolled molecular exchange across the nuclear membrane, thus adding further damage to the cells as documented by Sarikhani et al*.* [57]. Added to that, Cu is accused of provoking alterations in the chromatin pattern as mentioned by El-Samad et al. [58] and this could explain the peripheral chromatin condensation of the diseased cells in the present work.

The ultrastructural results of group II were in line with those of the light microscopic study. The degenerative changes were represented in the form of destructed architecture of the neurons. The heterochromatic nuclei indicated little or no transcriptional activity. According to Grewal [59], heterochromatin domains are rather less reachable for transcriptional machinery than euchromatin.

The current ultrastructural study of group II demonstrated obvious rarefaction of the cytoplasm, which was synchronized with the destructed architecture of the cells. In addition, there was an increase in the number of lysosomes, multivesicular, and alveolate bodies. As attributed to Ferrari et al. [60],the vital intracellular activities, including the breakdown of organelles and proteins, membrane repair, phagocytosis, and endocytosis might directly explain the rise in the number of those degrading structures.

The Intracellular cytoplasmic vacuolization seen in this study aligns with the findings of El Roghy et al. [61], who indicated that toxins and cytotoxic agents act as inducers of vacuolization. With a probable disturbance in the cell membrane, the intraorganellar osmotic pressure increases and the equilibration of osmotic pressure via water diffusion across organelle membranes leads to the creation of the vacuoles that accompany cell death [62].

The observed dilated rough endoplasmic reticulum and mitochondrial swelling were in line with the study of Elrashidy et al. [63] and El-Samad et al. [58]. The researchers attributed these changes to the up-regulation of endoplasmic reticulum stress markers and suppression of mitochondrial biogenesis-related proteins.

The observed neuronal death of group II might be significantly influenced by mitochondrial enlargement. From ATP generation to their critical role in cell death, mitochondria are involved in a variety of cellular processes. According to the current study, mitochondria might become uncoupled and greatly enlarged in response to oxidative stress, as reported by Rodríguez-Vera et al*.* [64]. In addition to this enlargement, prolonged pore opening brought on by extreme stressors also causes mitochondria-mediated cell death via necrosis or apoptosis [65]. This might be an additional explanation for the neuronal death seen in group II in the current investigation.

In the present study, the destructed myelinated nerve fibers of the neurons could be explained according to theories of Murumulla et al. [66]. The researchers thought that the alteration of the purinergic receptors of oligodendrocytes caused by metal toxicity resulted in a demyelinating process and thus fragmentation of myelin.

In contrast, coadministration of Cu and D-penicillamine (group III) reduced the observed histological alterations in the neurons of the caudate nucleus of group II. Tissue integrity is maintained by D-penicillamine to a degree that seems comparable to control. Haciyakupoglu et al. [67] claimed that this improvement could be related to the chelating properties of D-penicillamine, which reduce the amount of O_2_ radicals, inhibit inflammation, and remove transitional metals from the media. According to the investigators, D-penicillamine decreases the production of superoxide radicals and tissue damage by reducing iron and ferric proteins as well as binding and removing metal and metal complexes. Furthermore, it was postulated by the researchers that D-penicillamine inhibits lipid peroxidation, hence preventing vascular wall deterioration. Being of anti-inflammatory and anti-immunologic properties, it shields the nervous system and vascular wall from the inflammatory mediators that Cu releases.

Neuroglial cells are believed to be crucial for the growth, development, and preservation of myelin [50]. In response to damage, microglia use phagocytosis to remove misfolded proteins and myelin debris [68]. This could possibly facilitate the remyelination and could explain ultrastructurally the quiet normal appearance of the myelin figures.

## Conclusion

The current study provides novel evidence for the neuroprotective role of penicillamine in the caudate nucleus, a brain region critical for motor and executive functions. We showed that penicillamine treatment dramatically decreased oxidative stress, decreased inflammatory and apoptotic signals, and maintained structural integrity within the caudate using an in vivo model of CuSO_4_. These protective effects were further supported by improvements in the caudate structure, thereby linking biochemical and histopathological findings to functional relevance.

Crucially, the current study identifies a crucial research gap: although penicillamine has been thoroughly studied as a systemic chelator, nothing is known about how it acts in different regions of the basal ganglia, especially the caudate nucleus. The research provides mechanistic insight into how penicillamine modulates oxidative and inflammatory pathways at the striatal level. Looking forward and in order to enhance translational relevance, future research should test the long-term effects of penicillamine treatment in chronic models of caudate injury, assess dose–response and timing strategies for optimal neuroprotection, and incorporate behavioral and neuroimaging assessments. These guidelines will make it easier to understand penicillamine’s potential as a treatment for neurodegenerative and neurotoxic disorders including striatal dysfunction.

## Data Availability

The data that support the findings of this study are available upon reasonable request.

## Abbreviations

Cu: Copper
CuSO₄: Copper sulfate
GABA: Gamma-aminobutyric acid
H&E: Hematoxylin and eosin
H₂O₂: Hydrogen peroxide
IL-6: Interleukin-6
MDA: Malondialdehyde
TAC: Total antioxidant capacity
TBA: Thiobarbituric acid
TNF-α: Tissue necrosis factor-α
